# Effect of Heat Stress on Physiological and Behavioral Responses of Dehong Dairy Buffaloes

**DOI:** 10.3390/biology15080648

**Published:** 2026-04-20

**Authors:** Wei Huang, Fengyan Mei, Bin Deng, Jianping Ding, Xiqian Kuan, Zhiyong Cao, Xiujuan Yang

**Affiliations:** 1 Faculty of Agronomy and Life Sciences, Kunming University, Kunming 650214, China; huangwei_kmu@163.com; 2 Faculty of Animal Science and Technology, Yunnan Agricultural University, Kunming 650201, China; meifengyanynau@163.com (F.M.); dingjianping.ynau@edu.163.com (J.D.); kuanxiqian.ynau@edu.163.com (X.K.); 3 Yunnan Academy of Agricultural Engineering, Kunming 650215, China; 4 Yunnan Rural Science and Technology Service Center, Kunming 650021, China; dengbin_rc@163.com; 5 Faculty of Big Data, Yunnan Agricultural University, Kunming 650201, China; 6 Yunnan Key Laboratory Animal Nutrition and Feed Science, Yunnan Agricultural University, Kunming 650201, China

**Keywords:** heat stress, Denghong dairy buffaloes, physiological responses, behavioral responses, milk yield

## Abstract

This experiment aimed to evaluate physiological and behavioral responses of crossbred Dehong dairy buffaloes to heat stress (HS) in comparison with those in a thermoneutral (TN) environment. Twelve crossbred dairy buffaloes at similar lactation stages were randomly allocated to two groups of six animals each. Six buffaloes were exposed to HS conditions and the other six to TN conditions in an open loose-housing barn without individual stalls. Respiration rates were manually recorded at 08:00 h, 13:00 h, and 18:00 h. Duration and frequency of behaviors (standing, lying, feeding, and drinking) were continuously monitored using digital cameras for 20 consecutive days. Compared with the TN group, HS-exposed buffaloes exhibited markedly higher respiration rates (*p* < 0.001) and feeding frequencies (*p* < 0.05), but significantly shorter feeding duration throughout the observation period (*p* < 0.05). No significant differences were observed in the time spent standing, lying, or drinking between the two groups (*p* ≥ 0.05). Under HS conditions, buffaloes preferred a vertical lying posture to reduce exposure to intense solar radiation. These results suggest that crossbred Dehong dairy buffaloes can adapt to heat stress by modulating their physiological and behavioral strategies. The observed changes in physiological indices and behavioral patterns provide fundamental data for further elucidating the heat stress adaptation mechanisms in dairy buffaloes.

## Simple Summary

Buffaloes are important for producing milk, but hot and humid weather stresses them, hurting their health and milk output. Dehong dairy buffaloes, raised in Yunnan, China, face this problem in summer, yet we know little about how they adapt to heat. This study tested 12 similar lactating dairy buffaloes, six in hot stress conditions and six in thermal-neutral conditions. We checked their MY and composition, body temperature, respiratory rate, and behaviors like standing, lying, eating, and drinking. Under hot weather conditions, dairy buffaloes breathed faster, exhibited a higher feeding frequency but with shorter durations per feeding event, and tended to lie in a way that avoided direct sun; their body temperature was also slightly higher in heat stress conditions. We found that these dairy buffaloes adapt to heat stress via physiological and behavioral changes. Cooling strategies should be provided for Dehong dairy buffaloes to help reduce the harm caused by heat stress.

## 1. Introduction

Buffaloes (*Bubalus bubalis*) are the second most important source of raw milk globally, after dairy cows [1,2]. In Pakistan, buffaloes contribute approximately 68% of the national total milk production, compared with only 27% from cattle [3]. Additionally, buffalo milk has become the most preferred dairy product due to its higher fat and protein contents.

Dehong dairy buffaloes, as a swamp buffalo breed, are mainly distributed in Yunnan Province of China, which is geographically adjacent to Myanmar. With the aim of transforming production from meat/draught use to milk/meat dual-purpose production, swamp buffaloes (as female parents) have been artificially inseminated with frozen semen from river buffaloes (Murrah, Nili-Ravi) under a crossbreeding program supported by the European Union since 1997 [4]. Dehong Prefecture is the main breeding region for these crossbred dairy buffaloes, and its summer climate is characterized by high temperature and high humidity. The suitable ambient temperature for buffalo growth and reproduction is 13–18 °C, with a relative humidity of 55–65% [5]. Heat stress (HS) refers to a physiological imbalance in animals when internal heat production exceeds heat dissipation capacity, resulting in a series of adaptive and pathological responses. It is mainly caused by high ambient temperature, high humidity, strong solar radiation, poor ventilation, and high stocking density, which collectively disrupt the animal’s thermal homeostasis [6]. Dairy buffaloes are more sensitive to HS than dairy cattle due to their sparse hair, dark skin, and few sweat glands [7]. Buffaloes have insufficient sweating capacity and compensate by increasing their respiratory rate (RR) and panting; during summer with the highest ambient temperature and solar radiation, adult buffaloes cannot maintain thermal balance even when their respiratory frequency increases five to six times [8]. HS in livestock causes a series of serious production and health problems. Approximately 50% of milk production loss caused by HS is attributable to reduced dry matter intake, while the remaining 50% results from the direct physiological effects of HS [9]. Chronic HS impairs immune function, metabolic efficiency, and the overall health and welfare of buffaloes, leading to substantial economic losses in the dairy industry. Previous studies have shown that Dehong dairy buffaloes adapt to chronic HS partly by upregulating the expression of low-abundance serum proteins [10].

Exposure to intense solar radiation elevates body temperature and RR, which in turn reduces milk yield (MY), suppresses feed intake, retards growth performance, and lowers feed conversion efficiency, as commonly reported in heat-stressed dairy ruminants [11,12]. Under extreme high-temperature conditions, excessive heat load severely impairs animal health and welfare [13,14,15]. High-producing dairy buffaloes raised in hot and humid regions without adequate shade, wallowing, or swimming facilities show increased susceptibility to HS [16]. Consequently, stable milk production is seriously restricted by strong solar radiation and high temperatures in summer.

Animals can cope with HS through coordinated physiological and behavioral adaptive responses. However, systematic information regarding the physiological and behavioral adaptive strategies of Dehong dairy buffaloes under HS remains scarce. Therefore, the specific objective of this study was to evaluate the physiological and behavioral responses of crossbred Dehong dairy buffaloes to HS, including core body temperature, RR, panting score, and typical behavioral patterns such as standing, lying, and feeding. This research is of great practical significance for improving HS mitigation strategies, enhancing animal welfare, optimizing summer breeding and management practices, and ensuring the sustainable development of the local dairy buffalo industry.

## 2. Materials and Methods

### 2.1. Monitoring of Environmental Temperature and Evaluation of Heat Stress

Ambient temperature and relative humidity were measured at 30 min intervals using a Testo thermo-hygrometer. The temperature–humidity index (THI) was then computed using the following method:

Temperature and relative humidity of the air were measured using thermometers (±0.2 °C, Testo 175H1, Testo, Titisee-Neustadt, Germany) suspended 2.0 m above the floor of the bedding area at 30 min intervals for the calculation of THI as follows.

THI = (1.8 × T + 32) − [(0.55 − 0.0055 × RH) × (1.8 × T − 26)](1)

Note: T stands for air temperature (°C).

RH stands for relative humidity (%).

The occurrence of HS in dairy buffaloes was assessed using the environmental THI. HS was defined as a THI ≥ 72.

Based on meteorological data of two decades, meteorological data from 2010 to 2020 indicate that the summer THI exceeds 72, while the winter THI falls below 60. The average THI was above the comfort threshold (≥72) from June to August, and the average THI was above the comfort threshold (≥72) of dairy animals from June to August, and the average THI under thermoneutral conditions from January to February.

### 2.2. Experimental Animals and Management

The animals were handled in accordance with the regulations issued by the Animal Ethics Committee of Yunnan Province, China, in 2021. The experiment was conducted at a dairy buffalo farm in Ruili City, Dehong Prefecture, China, which lies within a South Asian subtropical monsoon climate zone (98.57° E, 24.43° N, at an elevation of over 870 m above mean sea level).

Based on the local climatic conditions, during June to August (average THI 75.76), 6 multiparous (parity = 3) Nili − Ravi × Murrah × local crossbred female buffaloes in mid-lactation (about 110–130 d) were used for the HS experiment (HS group) between 20 June and 20 August 2021, and other buffaloes with a similar parity and at a similar lactation stage were used for the thermoneutral experiment (TN group) and raised under TN conditions between January to February, 2022 (average THI 54.26, TN group).

Animals were housed in a loose-housing system with the same mixed rations (80% whole-plant corn silage, 12.5% feed concentrate, and 7.5% corn protein powder) and fresh drinking water provided ad libitum. The nutritional value of the feed is shown in Table 1.

### 2.3. Measurement of Vaginal Temperature

We referred to previous studies using blank controlled internal drug release (CIDR, DEC International NZ Ltd., Hamilton, New Zealand) devices to place a micro-temperature sensor (DS1922L, ±0.5 °C) to consecutively collect the vaginal temperatures of experimental animals at 30 min intervals [17,18].

### 2.4. Measurement Respiratory Rate

During the experiment, RRs were recorded when the buffaloes were at rest using a stopwatch at 08:00 h, 13:00 h, and 18:00 h. Chest movements were counted continuously for 3 min, and the count was converted to breaths per minute (breaths/min) to calculate a mean value. Each measurement was repeated three times, and the average was used for statistical analysis.

### 2.5. Observation of Dairy Buffalo Behaviors

Dairy buffalo behaviors observed included standing, lying, drinking and feeding, which were continuously observed for 20 days via high-definition cameras. The start and end times of each behavior bout were recorded to calculate the duration of each event. The total time was obtained by summing the durations of each behavior, and the total time was calculated by subtracting the total behavior time from the total observation time. The percentages of total observation time spent on each behavior were calculated separately.

The criteria for distinguishing each behavior are as follows [19]:

Standing: Four legs supported body weight;

Drinking: Muzzle keep in touch with water;

Feeding: At the feed trough visible feed is in the mouth;

Lying: Body flank keep in touch with ground. We speculated that dairy buffaloes may lie in optimal postures to reduce the impact of adverse ambient temperature, so we used “horizontal” and “vertical” to describe their lying postures. A “horizontal lying posture” means lying parallel to the axis of the animal house length, and a “vertical lying posture” means lying parallel to the axis of the animal house width.

### 2.6. Milk Yield and Milk Composition of Dairy Buffaloes

MY was determined using portable milking machines. During the formal trial period, measurements were obtained every two days, with MY recorded three times daily (morning, noon, and evening), the sum of which was taken as the daily milk yield for that day.

Milk composition analysis: Milk samples from the experimental cows were collected using a flow meter, and an online milk composition analyzer (FOSS Combi FT + FC, Hillerød, Denmark) was used to measure the butterfat percentage, milk protein percentage, somatic cell count (SCC), urea nitrogen, and lactose percentage in the milk.

### 2.7. Statistical Analysis

Standing and lying behavior data were sequentially extracted for each dairy water buffalo from the video files. Microsoft Excel 2010 was used to integrate and process the behavioral data. Physiological and behavioral parameters were obtained for individual animals, after which the group-level data were combined. Independent samples *t*-tests were employed to analyze parameters including body temperature and RR.

## 3. Results

### 3.1. Air Temperature and THI Indicated of Buffaloes

**Figure 1:**
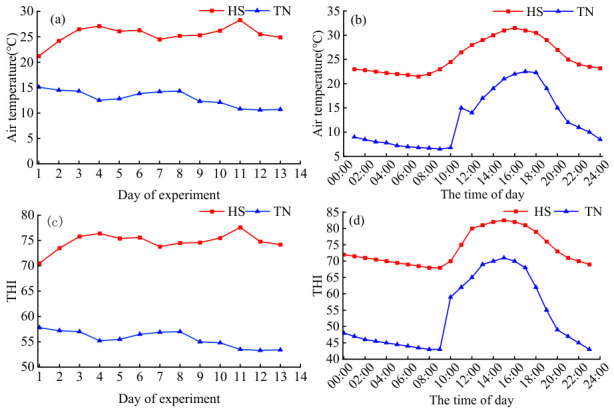
Rhythms of the indoor air temperature and THI for buffaloes: (**a**) the average indoor air temperatures of the experimental day, (**b**) the circadian rhythm of temperature, (**c**) the average THI of the experimental day, and (**d**) the circadian rhythm of the THI. HS, Heat stress buffaloes (red). TN, Thermal-neutral buffaloes (blue).

Under heat stress conditions, the mean indoor air temperature on experimental days exceeded 25 °C, whereas it was below 15 °C under non-heat-stress (thermoneutral, TN) conditions (Figure 1a). With regard to the diurnal rhythm of temperature, temperatures were >25 °C from 10:00 h to 19:00 h and >30 °C from 12:00 h to 17:30 h under heat stress (HS) conditions (Figure 1b). Similarly, the average indoor THI was mostly above 72 under HS conditions, and THI values exceeded 72 for 21 h out of 24 h (Figure 1c,d). In contrast, all THI values were below 67 under TN conditions. The elevated temperatures and THI values demonstrated that Dehong dairy buffaloes experienced HS during summer.

### 3.2. Milk Yield and Milk Composition of Dairy Buffaloes

**Figure 2:**
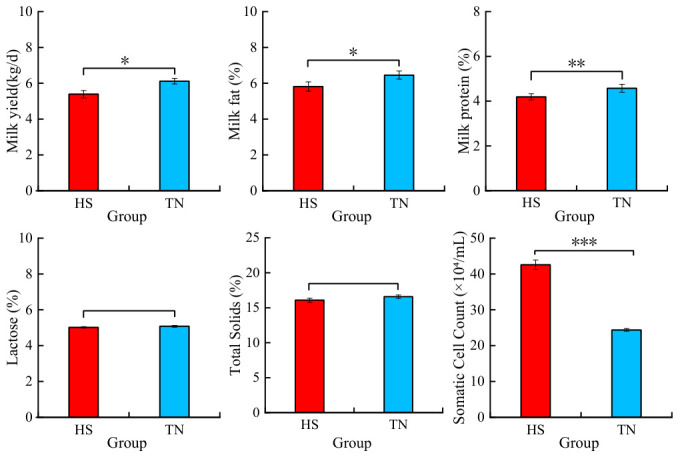
Milk yields and milk composition of the HS and TN groups for dairy buffaloes. Note: For comparison between two groups, no asterisk indicates no significant difference, one asterisk indicates a 5% significance level (*p* < 0.05), two asterisks indicate a 1% significance level (*p* < 0.01), and three asterisks indicate a 0.1% significance level (*p* < 0.001). HS, Heat stress dairy buffaloes (red). TN, Thermal-neutral dairy buffaloes (blue). The same as below.

HS influenced the daily milk yield (MY) and milk composition of Dehong dairy buffaloes (Figure 2). Buffaloes under HS exhibited lower MY compared with the TN group (*p* < 0.01). HS significantly reduced milk fat percentage (*p* < 0.05), markedly decreased milk protein percentage (*p* < 0.01), and highly significantly reduced SCC (*p* < 0.001). No significant effect was observed on total solid percentage and total solid content (*p* ≥ 0.05), although both variables exhibited a decreasing tendency.

### 3.3. Physiological Parameters of Dairy Buffaloes

**Figure 3:**
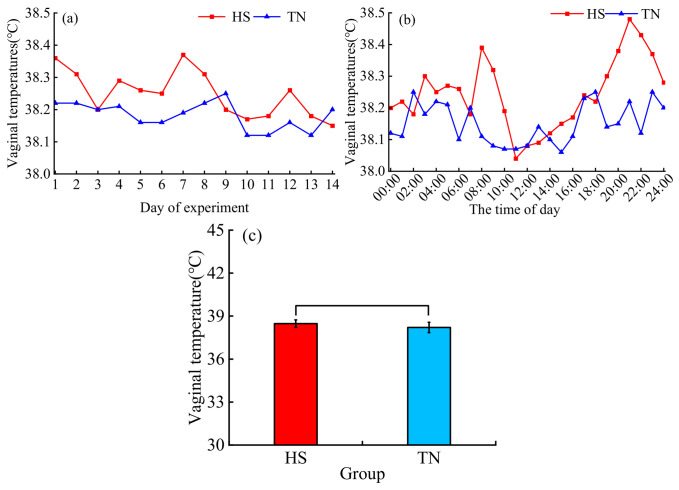
Vaginal temperatures for buffaloes: (**a**) the average vaginal temperature of the experimental day; (**b**) the circadian rhythm of vaginal temperature; (**c**) the average vaginal temperature throughout the entire experiment period. HS, Heat stress buffaloes (red). TN, Thermal-neutral buffaloes (blue).

Buffaloes under HS exhibited higher body temperatures (assessed by vaginal temperature) compared with the TN group (Figure 3a). Body temperatures in the HS group were higher than those in the TN group from 08:00 h to 09:00 h and from 18:30 h to 22:00 h. No significant differences in body temperature were observed between the HS and TN groups from 02:30 h to 07:30 h and from 10:00 h to 17:00 h (Figure 3b). Similarly, there were no significant differences in average body temperature between the two groups (Figure 3c).

**Figure 4:**
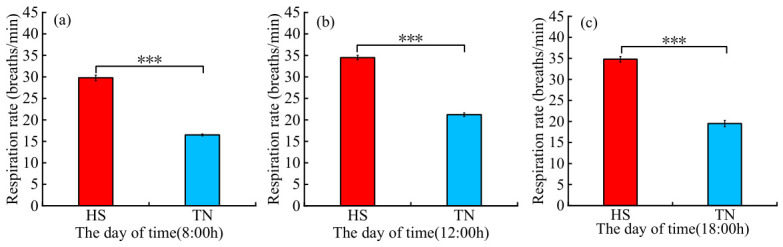
Respiration rate of the HS and TN groups for dairy buffaloes. (**a**) the respiration rate measured at 8:00 h; (**b**) the respiration rate measured at 12:00 h; (**c**) the respiration rate measured at 12:00 h. HS, Heat stress buffaloes (red). TN, Thermal-neutral buffaloes (blue). Note: Three asterisks indicate a 0.1% significance level (*p* < 0.001).

As shown in Figure 4, the respiration rate of the HS group was significantly greater than that of the TN group (*p* < 0.001) at 08:00 h, 13:00 h and 18:00 h.

### 3.4. Behaviors of Dairy Buffaloes

The behavioral patterns of Dehong dairy buffaloes are summarized in Table 2. No significant differences were observed in the time budgets for standing, lying, and drinking between the HS and TN groups.

Similarly, no significant differences were detected in the durations of standing, lying, and drinking. However, the duration of feeding in the HS group was significantly shorter than that in the TN group.

**Figure 5:**
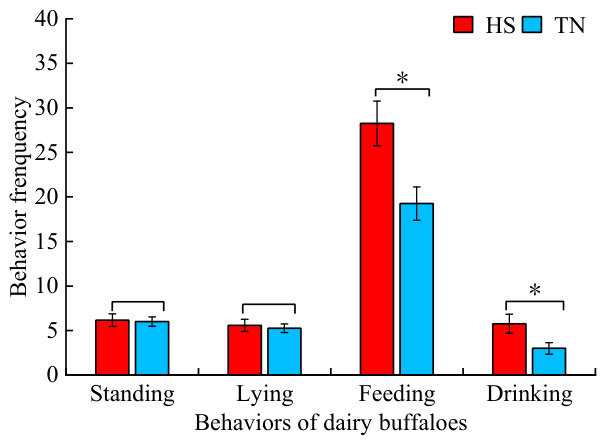
Behavior frequencies of the HS and TN groups for buffaloes. Note: For comparison between two groups, no asterisk indicates no significant difference, one asterisk indicates a 5% significance level (*p* < 0.05). HS, Heat stress dairy buffaloes (red). TN, Thermal-neutral dairy buffaloes (blue).

As shown in Figure 5, no significant differences were detected in standing and lying frequencies between the HS and TN groups; however, the HS group exhibited significantly different feeding and drinking frequencies compared with the TN group.

### 3.5. Lying Behaviors of Buffaloes

**Figure 6:**
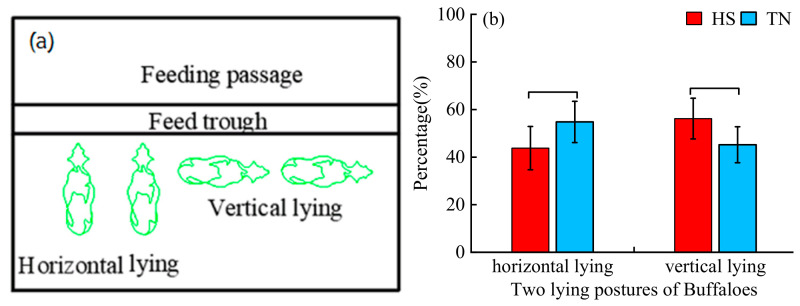
Buffalo lying behaviors in two postures. (**a**) Posture for Buffaloes: Horizontal lying means lying parallel to the axis of the animal house length, and vertical lying means lying parallel to the axis of the animal house width. (**b**) Lying posture percentage of HS and TN groups for buffaloes. HS, Heat stress dairy buffaloes (red). TN, Thermal-neutral dairy buffaloes (blue). Note: For comparison between two groups, no asterisk indicates no significant difference.

The two lying postures were horizontal lying (parallel to the length axis of the animal house) and vertical lying (parallel to the width axis of the animal house). Due to the limited bedding area (2.4 m) in the open barn, we hypothesized that dairy buffaloes under HS would spend a higher percentage of lying time in the vertical posture compared with the horizontal posture. In fact, the percentage of vertical lying time (56.2%) was higher than that of horizontal lying time (43.8%) in the HS group; however, no significant difference was detected between these two postures. Similarly, in the TN group, the percentage of horizontal lying time (54.8%) was higher than that of vertical lying time (45.2%) (Figure 6).

No significant differences were observed in the time budgets for the two lying postures between the HS and TN groups. Although no significant difference was detected, the percentage of vertical lying time in the HS group (56.2%) was slightly higher than that in the TN group (45.2%).

## 4. Discussion

Heat stress (HS) represents a widespread environmental stressor that exerts adverse effects on milk production, reproductive performance, and overall health of dairy ruminants [20]. For Holstein cattle, the THI above 72 is closely associated with significant reductions in daily MY under automated milking systems, and exposure to a THI ranging from 75 to 87 induces marked changes in blood metabolites during early and mid-lactation [21,22]. In the present study, severe HS was clearly confirmed by the measured environmental parameters. During the HS period, the average indoor ambient temperature exceeded 25 °C, and the indoor THI exceeded 72 for up to 21 h per 24 h period; in contrast, THI values remained below 67 under thermoneutral (TN) conditions. Significant correlations were also observed between barn environmental indices and the physiological indicators of dairy buffaloes [23]. As a reliable and effective indicator for assessing HS intensity in dairy livestock, THI has been widely used in evaluating the thermal comfort of dairy animals [24]. The high ambient temperature (>25 °C) and elevated THI (>72) observed in this study indicated that Dehong dairy buffaloes were unable to maintain normal thermal homeostasis.

Compared with cattle, dairy buffaloes have underdeveloped sweat glands and a weaker cutaneous heat dissipation capacity. In addition, buffaloes typically have black skin and a sparse hair coat, which make them more prone to absorbing solar radiation and accumulating body heat under hot environmental conditions. Together, these anatomical characteristics render dairy buffaloes particularly susceptible to HS compared with other dairy ruminants [25]. In dairy cattle, evaporative heat loss via the respiratory tract accounts for only approximately 15% of total heat dissipation [26]. Under HS, buffaloes typically respond by increasing body temperature and RR to enhance evaporative heat loss [27]. Moreover, the experimental site is characterized by high temperatures and high humidity in summer. Such hot, humid conditions markedly impair heat dissipation and disrupt physiological homeostasis in buffaloes. When the ambient temperature exceeds 30 °C, high RH severely reduces evaporative cooling efficiency and further exacerbates heat load accumulation. Consistent with observations in tropical climates, elevated RH and surface temperature act as key adaptive responses to thermal challenge; yet, their effectiveness is greatly diminished under intense heat and humidity [28]. Taken together, the persistent high temperatures and high THI in summer induced obvious HS in Dehong dairy buffaloes. The inefficient thermoregulatory capacity, characterized by limited sweating and reliance on panting, combined with high humidity restricting evaporative heat loss, collectively rendered Dehong dairy buffaloes highly susceptible to thermal stress, thereby disrupting their normal physiological homeostasis.

Under this chronic HS condition, Dehong dairy buffaloes exhibited significant decreases in MY, milk fat percentage, milk protein percentage, and SCC, accompanied by a non-significant downward trend in total solids (optimized phrasing for SCI rigor). These alterations in production performance are consistent with the mammary dysfunction induced by HS reported in dairy cattle and other buffalo breeds, although species-specific differences in thermoregulatory capacity may contribute to variations in the magnitude of these responses. In dairy cows, HS has been demonstrated to alter the composition and viability of somatic cell populations in milk, including increasing mammary epithelial cell shedding and reducing the number of viable immune cells; this not only compromises mammary secretory function but also weakens local immune competence in the mammary gland [29]. Similarly, HS suppresses dry matter intake and downregulates the expression of key genes associated with milk synthesis in mammary cells, thereby directly reducing MY in crossbred dairy cows [30]. In the present study, the simultaneous decline in MY, milk fat, and milk protein in heat-stressed buffaloes further supports impaired synthetic activity in mammary epithelial cells. Meanwhile, the significant reduction in SCC observed in buffaloes differs somewhat from those reported in certain indicine crossbred cattle, which may reflect distinct immune and cellular shedding patterns in the buffalo mammary gland under thermal challenge. Collectively, these production changes reflect a general disruption of mammary homeostasis and synthetic function elicited by chronic HS.

Previous studies have reported that buffaloes are more sensitive to HS than other domestic ruminants [2]. For instance, when the body temperatures of lactating buffaloes increased from 37.9 °C to 39.7 °C, their respiratory rates increased correspondingly from 23.4 to 41.0 breaths/min [31]. In the present study, HS-exposed dairy buffaloes exhibited numerically higher vaginal temperatures compared with the TN group, with elevated body temperatures observed during the periods of 08:00–09:00 h and 18:30–22:00 h. However, no significant differences were detected in overall body temperature between the two groups. Despite the persistent thermal load imposed by chronic heat exposure, Dehong crossbred dairy buffaloes did not exhibit a statistically significant elevation in body temperature. We therefore hypothesize that Dehong crossbred dairy buffaloes may have gradually acclimated to chronic HS through coordinated behavioral and physiological thermoregulatory responses, thereby enabling them to maintain relative thermal homeostasis under prolonged high-temperature conditions.

HS is well known to induce extensive alterations in behavioral and physiological responses in dairy ruminants [32]. In the present study, most general behavioral patterns, including standing, lying, and drinking time, remained largely unaffected between heat-stressed and thermoneutral conditions. This relative stability in general activity may reflect the strong adaptive capacity of Dehong dairy buffaloes to chronic thermal environments, allowing them to maintain basic behavioral rhythms under moderate thermal load.

By contrast, feeding duration was significantly decreased under HS. This response is consistent with the general thermoregulatory strategy observed in dairy cattle, in which animals voluntarily reduce feeding time to minimize the metabolic heat increment associated with feed digestion and fermentation [33,34]. Reducing feed intake effectively lowers internal heat production, thereby alleviating thermal burden without excessive dependence on evaporative cooling. This reduction may be mediated by HS-induced inhibition of the lateral appetite center in the hypothalamus, as well as the need to limit heat derived from rumen fermentation that must be dissipated [35]. Although drinking frequency tended to increase in heat-stressed buffaloes, drinking time did not differ significantly, which may be attributed to the high moisture content of the diet, such as fresh maize straw and green forage, thereby reducing the requirement for supplementary drinking water. In terms of lying postures, heat-stressed buffaloes showed a numerical trend toward more frequent vertical lying compared with animals under thermoneutral conditions. This behavioral pattern can be interpreted as a passive adjustment to confined bedding space and intense ambient solar radiation. By adopting a vertical lying posture, buffaloes may reduce body surface exposure to radiant heat and enhance convective heat loss around the body. However, the lack of statistical significance indicates that this adjustment is mild and context-dependent, rather than a dominant thermoregulatory behavior. Nevertheless, frequent vertical lying in restricted space may compromise resting comfort and disrupt normal social lying patterns, which could indirectly impair animal welfare and long-term productivity under sustained HS.

Dehong is located in the southern subtropical monsoon climate zone of China, which provides natural climatic conditions for conducting experiments on heat stress and thermal neutrality in Dehong buffaloes. For large animals such as cattle, specialized climate chambers are required for thermal neutrality experiments, and the cost of constructing such new facilities is approximately 700,000 to 800,000 RMB. In contrast, this experiment can be implemented with reliance on the local natural environment, effectively avoiding additional facility investment. To minimize the interference of seasonal confounding factors, the temperature–humidity index (THI) was used as a comprehensive environmental evaluation indicator to reduce the impact of complex environmental factors on the experimental results to the greatest extent. The obtained findings can truly reflect the environmental adaptation patterns of Dehong buffaloes under natural heat stress conditions, which possess important practical reference value and ecological application significance for the research and utilization of this excellent local breed.

## 5. Conclusions

In summer, Dehong dairy buffaloes exhibit a decrease in milk yield, milk fat content, milk protein content, and somatic cell count, while increasing heat dissipation by elevating body temperature, respiratory rate, and water intake duration. Under heat stress, feeding frequency increases, whereas feeding time decreases in dairy buffaloes. In addition, buffaloes under heat stress adopt a vertical lying posture to avoid direct solar radiation. These behavioral and physiological adjustments—including elevated respiratory rate, modified feeding patterns, and vertical lying—enable buffaloes to modulate their physiological and behavioral responses to adapt to heat stress. Cooling strategies should be implemented to mitigate the significant adverse impacts of chronic heat stress on dairy buffaloes in tropical and subtropical regions during summer.

## Figures and Tables

**Table 1:** Feed nutritional value.

Item	Whole-Plant Corn Silage	Concentrate Feeding	Corn Protein Powder
Fresh matter (%)			
Moisture	73.64	8.65	8.3
CF	1.48	4.14	1.17
ASH	5.71	21.31	3.66
CP	7.56	40.18	67.22
Ca	0.3	3.29	0.18
P	0.2	1.08	0.54
Dry matter (%)			
ADF	35.25	15.48	8.97
ADICP	0.35	4.06	11.56
NDF	55.26	20.63	20.74
NDICP	0.24	6.18	5.98
ADL	4.95	4.34	3.3
NFC	30.24	19.92	13.19
tdNFC	29.63	19.52	12.93
tdNDF	30.97	3.15	4.84
tdCP	7.15	35.59	54.69
tdFA	0.48	3.14	0.17

**Table 2:** Time percentages (%) and duration (min) of observed behaviors of dairy buffaloes (mean ± S.E).

	Time Percentages (%)		Duration (min)	
	HS	TN	HS	TN
Standing	56.7 ± 5.2 ^a^	54.8 ± 1.9 ^a^	59.7 ± 5.7 ^a^	58.0 ± 4.2 ^a^
Lying	43.3 ± 5.2 ^a^	45.2 ± 1.9 ^a^	58.0 ± 10.6 ^a^	55.8 ± 6.1 ^a^
Feeding	16.4 ± 1.6 ^a^	23.8 ± 1.5 ^b^	3.9 ± 0.6 ^A^	8.7 ± 1.4 ^B^
Drinking	0.5 ± 0.2 ^a^	0.4 ± 0.1 ^a^	0.5 ± 0.2 ^a^	0.7 ± 0.2 ^a^

Note: In the same row, values with the same letter superscripts mean no significant difference (*p* > 0.05), different lowercase superscript letters indicate a significant difference (*p* < 0.05), and different uppercase letters indicate extremely significant differences (*p* < 0.01).

## Data Availability

The raw data supporting the findings of this study are available from the corresponding author on reasonable request due to institutional ethical restrictions regarding animal experiment data sharing.

## Abbreviations

The following abbreviations are used in this manuscript:

DMI	Dry matter intake
HS	Heat stress
MY	Milk yield
RR	Respiratory yate
RH	Relative humidity
SCC	Somatic cell count
THI	Temperature–humidity index
TN	Thermal-neutral
